# Category bias in similarity ratings: the influence of perceptual and strategic biases in similarity judgments of faces

**DOI:** 10.3389/fcogn.2023.1270519

**Published:** 2023-11-15

**Authors:** Stefania R. Ashby, Benjamin Chaloupka, Dagmar Zeithamova

**Affiliations:** 1 Department of Psychology, Brigham Young University, Provo, UT, United States; 2 Neuroscience Center, Brigham Young University, Provo, UT, United States; 3 Department of Psychology, University of Oregon, Eugene, OR, United States

**Keywords:** category learning, perceptual bias, strategic judgment bias, similarity-based categories, arbitrary categories

## Abstract

**Introduction:**

After category learning, same-category items tend to be rated as more similar than items from different categories. Whether this category bias in similarity ratings reflects true changes in perception or a strategic judgment bias to rate same-category items more similarly has been debated. The current study investigated the influence of perceptual and strategic judgment biases on perceived similarity ratings of face stimuli.

**Method:**

To explore the influence of perceptual and strategic biases, post-learning category bias was measured after learning one of two category structures. In a similarity-consistent structure, faces within a category shared physical features and category bias could reflect a combination of strategic bias and true perceptual changes. In a similarity-inconsistent structure, category membership was orthogonal to physical features and category bias could only be driven by strategic bias to rate same-label faces as more similar.

**Results:**

We found a strong category bias after learning, but only when category labels could be aligned to the similarity structure. When category label conflicted with similarity structure, the mere presence of a shared label did not create a bias.

**Discussion:**

These findings indicate that category bias in this paradigm is largely driven by a perceptual bias, consistent with proposals that category learning can stretch or shrink perceptual space by biasing attention toward category-relevant and away from category-irrelevant features. More broadly, these findings contribute to our understanding of category-driven biases and may inform bias research in other domains such as social stereotypes.

## Introduction

We encounter a massive amount of information daily. Categorization is one of the ways we organize this plethora of data for future use. Category knowledge allows us to organize that information into meaningful bundles and generalization of category knowledge guides our decisions in novel situations (Markman and Ross, 2003; Ashby and Maddox, 2005, 2011; Seger and Miller, 2010; Goldstone et al., 2018). Category knowledge also affects perception: items within a category are viewed as more similar while items from different categories are viewed as less similar (see Livingston et al., 1998; Goldstone and Steyvers, 2001). This category bias in perception has been documented for well-established categories such as discriminating hues across color boundaries (Bornstein et al., 1976; Sandell et al., 1979; Bornstein and Korda, 1984; Franklin and Davies, 2004; Winawer et al., 2007) and discriminating speech sounds across phonemic boundaries (Liberman et al., 1957). Even categories like race induce a category bias in perception (Levin and Angelone, 2002) which may in part explain other phenomena like own-race bias where individuals are better at differentiating people of their same race (Timeo et al., 2017). Additionally, category biases in perception can emerge after individuals learn new categories in the laboratory (Rosch and Mervis, 1975; Goldstone, 1994a,b; Beale and Keil, 1995; Goldstone et al., 2001), and can be induced by the presence of a shared category label even when task instructions emphasize memory for individual items (Ashby et al., 2020; Ashby and Zeithamova, 2022; Bowman et al., 2022).

In lab studies examining the impact of newly learned categories, the presence of a category bias in perception has commonly been studied using perceived similarity ratings (Goldstone, 1994a; Beale and Keil, 1995; Kurtz, 1996; Livingston et al., 1998). Typically, participants are shown a set of stimuli and asked to rate how alike all stimuli are to one another. These ratings are taken prior to category learning and then again after participants have learned category information. Evidence for category bias in these similarity ratings is reflected by greater perceived similarity ratings after learning for items that belong to the same category (Goldstone, 1994a; Levin and Beale, 2000; Ashby et al., 2020), and/or lower similarity ratings for items that belong to different categories (Kurtz, 1996; Livingston et al., 1998; Ashby et al., 2020). Our prior work has examined category bias in perceived similarity ratings after participants learned a family category structure of face-blend stimuli varying along many continuous dimensions. We have shown that a category bias in perceived similarity ratings of faces can arise in brain and behavior from the mere presence of a shared family label even when individuals are not explicitly aware of an underlying family category structure and task goals focus on memorizing individual faces rather than category learning (Ashby et al., 2020; Ashby and Zeithamova, 2022). Furthermore, the categorization bias predicts how well individuals can generalize category information to new faces in both young adults (Ashby et al., 2020; Ashby and Zeithamova, 2022) and older adults (Bowman et al., 2022), suggesting that it tracks category knowledge.

What drives changes in perceived similarity ratings following category learning? The mechanism responsible for category bias has been debated. Some have postulated that category learning affects how we perceive the world by warping perceptual space. As attention is biased toward category-relevant features and away from category-irrelevant features, the perceptual space stretches along the relevant dimensions and shrinks along the irrelevant dimensions, making within-category similarity and between-category differences more apparent (Nosofsky, 1984, 1986; Kruschke, 1992, 1996; Lupyan, 2012). Thus, under this perceptual bias account, category learning alters representations of individual items to align with category membership. In support of this account, past behavioral work has indicated that information is more discriminable across category-relevant dimensions after category learning (Goldstone, 1994b; Goldstone and Steyvers, 2001; Gureckis and Goldstone, 2008). Further, increased discriminability of items on a category-relevant dimension has been shown to extend to more complex category structures (e.g., object shape and object motion dimensions; Folstein et al., 2014) and result in larger increases in within-category similarity after learning compared to more simple category structures (Pothos and Reppa, 2014).

Neuroimaging work has provided additional support. For example, Folstein et al. (2013b) indicated that better ability to discriminate among category-relevant features after learning was associated with increased discriminability of their neural representations in visual cortex. Likewise, Juárez et al. (2019) found that an increase in the N1 neural response—typically associated with early perceptual processing in category learning (for example see Curran et al., 2002)—predicted a decrease in similarity ratings for items that belong to different categories after learning. And recent work from our lab found evidence for category-biased neural representations widespread across the cortex that emerged during learning, even when physical similarity was controlled for and category information was not explicitly emphasized (Ashby and Zeithamova, 2022). Taken together, we have proposed that changes in perceived similarity ratings after category learning in our prior work reflect true changes in perception as attention is shifted toward category-relevant and away from category-irrelevant features to better align perceived similarity with category membership.

However, category biases may not just reflect changes in perceptual space. For example, Goldstone and colleagues (2001) suggest an alternative to the perceptual change hypothesis asserting that biases in perceived similarity ratings may also reflect *strategic judgment* bias to take the category label into account when rating similarity. In other words, one may provide higher ratings of items from the same category and lower ratings of items from different categories simply because their same vs. distinct category labels are considered, rather than because they appear more or less similar. Indeed, a contribution of the strategic judgment bias to perceived similarity ratings was concluded in a study in adult category learners by Goldstone and colleagues (2001) which included new items not previously associated with a category label during the similarity rating phase. This also aligns with other work showing that introducing verbal interference disrupts learning of shared linguistic labels which abolishes categorical perception effects (Roberson and Davidoff, 2000; Pilling et al., 2003; Hanley and Roberson, 2011; Simanova et al., 2016) and developmental work showing an effect of shared linguistic labels on similarity judgments in children (Sloutsky and Lo, 1999; Booth, 2014). Thus, differentiating between a perceptual bias and a strategic judgment bias account is important for a mechanistic understanding of the role of concepts in cognition and perception.

Having found strong category biases in brain and behavior in our prior work using a face-blend category learning task (see Ashby et al., 2020; Ashby and Zeithamova, 2022; Bowman et al., 2022), the current study sought to determine the degree to which perceptual and strategic judgment bias may contribute to the similarity ratings measures in this paradigm. We reasoned that when faces belonging to the same category share features (as they typically do), category bias in perceived similarity ratings may be fully or partially driven by true changes in perception, such as through attentional bias toward category-relevant features. On the other hand, when category labels are arbitrary and faces within categories do not share features, one cannot alter within-category similarity or between-category differences through learned attentional biases. As such, any change in perceived similarity ratings would be driven by a strategic judgment bias to rate same category faces similarly. By comparing similarity rating bias observed under these two conditions, we can determine the degree to which strategic judgment bias contributes to category bias in post-learning perceived similarity ratings.

Using a traditional feedback-based category learning task and face-blend stimuli developed in our prior work to control physical similarity of naturalistic stimuli (Ashby et al., 2020; Ashby and Zeithamova, 2022; Bowman et al., 2022), we tracked post-learning category bias under two category conditions. Some participants learned categories where stimuli within a category shared features while others learned categories where stimuli within a category did not share features. We reasoned that if category bias in similarity ratings is more reflective of a strategic judgment bias, then category bias in similarity ratings would be expected after learning in both category conditions, even when participants learn category labels that are orthogonal to the physical similarity structure of the stimuli. Alternatively, if a category bias in ratings is more reflective of changes in perception or attention to category-relevant features, then a category bias in perceived similarity ratings would only be expected following learning of categories based on shared features. And finally, if category bias in similarity ratings is a combination of true changes in perception and a strategic judgment bias, comparing the degree of category bias between conditions will allow us to estimate the relative contribution of each process to the total category bias.

## Methods

### Data transparency and openness

We report how we determined our sample size, all data exclusions, all manipulations, and all measures in the study. The design and analysis plan for this experiment were preregistered via the Open Science Framework (OSF; https://osf.io/9serh) and all data and materials are also publicly available via OSF (https://osf.io/bhc32/).

### Participants

Two-hundred and 15 participants were recruited from the University of Oregon via the university SONA research system and completed the study online via Pavlovia (www.pavlovia.org) in 2020. Participants received course credit as compensation for their participation. All participants provided informed consent and experimental procedures were approved by Research Compliance Services at the University of Oregon. Six participants were excluded from analyses for failing to respond to 20% or more of the trials in either the similarity ratings or category learning phases. The remaining sample of 209 participants are reported in all analyses. Due to experimenter error in pivoting to online data collection amidst the COVID-19 pandemic, demographic information was only collected for approximately half the sample (93 female, 19 male, 3 unreported; age 18–34 years; M_age_ = 19.46, SD_age_ = 2.05). Since all participants were recruited through introductory psychology courses at the University of Oregon, we are confident that the limited demographic information collected is representative of the overall sample.

#### Rationale for sample size

The present study compared the category bias effect sizes between two groups of subjects, one that learned similarity-based categories (similarity-consistent condition) and one that learned categories that lacked a similarity structure (similarity-inconsistent condition). Sample sizes of at least 100 participants per condition were determined *a priori* via power analyses in G*Power (Faul et al., 2007, 2009) utilizing effect sizes from previous studies (see Ashby et al., 2020; Ashby and Zeithamova, 2022; Bowman et al., 2022). Prior data demonstrated that when categories were similarity-consistent, the effect of a category bias in similarity ratings post-learning was medium-sized (Cohen’s *d* = 0.51). We reasoned that this effect size may be reflective of a combination of perceptual change and strategic processes in the similarity-consistent condition, with at least one of these effects contributing at least half (*d* = 0.25) of the total effect size. In contrast, no true perceptual changes would be expected in the similarity-inconsistent condition, and any category bias in similarity ratings would be due solely to strategic processes (with effect size expected to be much smaller than *d* = 0.5). Thus, our target was 80% power to detect a small effect (*d* = 0.25, or half of the total *d* = 0.5 expected in the similarity-consistent condition based on our prior data) at the standard alpha error probability level (α = 0.05). This target is sufficient for both detecting differences between conditions and detecting a presence of category bias in the similarity-inconsistent condition (if one exists).

### Stimuli

Stimuli were grayscale images of blended faces created by morphing pairs of “parent” faces (FantaMorph Version 5 by Abrosoft) originating from the Dallas Face Database (Minear and Park, 2004), the Computer Vision Laboratory Database (Peer, 1999), and a Google Image search (Figure 1). Ten parent faces were selected for blending from a larger pool of faces used in prior work (see also Ashby et al., 2020; Ashby and Zeithamova, 2022; Bowman et al., 2022). We found in previous data that face blends of very distinctive parent faces were dominated by features of those parents, while features of more average parent faces tended to be lost in the resultant face-blend. Thus, we selected 10 faces that were neither too distinctive nor too average, helping us ensure that all face blends were about equally physically similar to both parent faces rather than randomly biased toward one parent or the other. From the set of 10 parent faces, six were randomly selected for each participant and blending was conducted by morphing three of these parent faces (“A” Parents) with the remaining three parent faces (“B” Parents) individually. This resulted in a set of nine face-blends that shared 50% features from each parent to be used in category learning and similarity ratings tasks (see Figure 1). To measure changes related to category learning and not mere differences in exposure frequency to different face features, it was important that the training set was derived using an equal number of “A” and “B” parent faces. The 3x3 stimulus structure ensured that the task was not trivially easy (as would be the case with 2x2 structure) while resulting in a manageable number of pair-wise comparisons among all stimuli for the subsequent similarity rating task. To minimize any potential item effects in similarity ratings, participants were yoked within and across conditions, such that for each participant in the similarity-consistent condition with a given set of “A” and “B” parents, there was a participant in the similarity-inconsistent condition with the same parent-face selection, as well as one participant in each condition using the same parent faces but with reversed “A” and “B” parent assignment. The original, nonblended parent faces were never shown to participants nor used during any stage of the experiment.

Using blended faces allowed us to generate more naturalistic, realistic-looking stimuli that vary along many ill-defined features, akin to what we frequently encounter in everyday life, while still controlling their physical similarity. Our prior work demonstrated that participants are highly sensitive to the similarity structure introduced by face blending, such that two faces that share a parent (and thus exhibit that parent’s facial features) are perceived as much more similar than two faces that do not share a parent (e.g., Cohen’s *d* = 1.50 in pre-learning similarity ratings in Ashby et al., 2020). Furthermore, we used the same stimuli and category structure as our prior work in order to better discern among competing interpretations of our previous work in light of findings from the current study.

Because we were interested in biases driven by the category learning task, it was important that the blended faces were not easily sorted into pre-existing categories prior to training on face families. Because race and gender are their own preexisting categories, all parent faces had to be of a single race and single gender. We chose white faces for the stimulus set because prior work has shown that white Americans exhibit greater own-race effects in face recognition than non-white Americans (Meissner and Brigham, 2001; Herzmann et al., 2017), and we expected our sample to be majority white based on the University of Oregon population demographics (64% white). We chose male for the face gender because females show less own-gender bias than males (Mishra et al., 2019) and because female faces have a greater variability in hair styles that can make the blending process difficult. Exploratory analyses of data from our prior work (see Ashby et al., 2020) indicate that category learning and the resulting category bias in this paradigm do not differ between male and female participants.

### Experimental conditions

Participants were randomly assigned into one of two experimental conditions where they learned to sort the nine face-blends into three categories labeled by family name (Miller, Wilson, Davis). The experimental conditions participants were assigned to determined how the face-blends were assigned into family categories either based on a shared parent (similarity-consistent) or a lack of a shared parent (similarity-inconsistent).

#### Similarity-consistent family structure

For similarity-consistent categories, family assignment was aligned with the physical similarity among faces (Figure 1A). Specifically, three of the parent faces (“A” Parents) determined the three family categories (Miller, Wilson, Davis). Any face-blend of that parent face was considered a member of the same category. Because they were blended from the same parent, face-blends from a given category not only shared a family name but also shared physical features (see faces across rows in Figure 1A). Notably, faces that shared a “B” parent also shared physical features but belonged to different families (see faces down columns in Figure 1A). Thus, physical similarity alone did not determine category membership and participants needed to learn which face features were relevant to category membership through training. Category bias was measured by comparing similarity ratings for pairs of faces that shared a parent and belonged to the same family category with similarity ratings for pairs of faces that also shared a parent but belonged to different categories (rows vs. columns in Figure 1A). A tendency to rate faces that share category-relevant “A” parents as more similar than those that share category-irrelevant “B” parents would constitute a category bias in perceived similarity rating. We reasoned that this category bias would be a combination of a true perceptual bias and a strategic judgment bias (total bias = perceptual bias + strategic judgment bias).

#### Similarity-inconsistent family structure

For similarity-inconsistent categories, the same blended faces were used but family assignment was orthogonal to the physical similarity of the face-blends, such that faces from the same category shared a family name but critically did **not** share a parent face (Figure 1B). Thus, there were no relevant or irrelevant physical features to be extracted for the categories and instead learning required that the category label be memorized for each face. This category structure is conceptually similar to the Type 6 category structure from seminal work by Shepard et al. (1961). Category bias can still be measured by comparing ratings for faces that do not share a parent but belong to the same category vs. those who do not share a parent and belong to different categories (i.e., faces that belong vs. not belong to the same category, controlled for physical similarity). Because the stimuli within a category do not share features, learning is unlikely to lead to true perceptual biases and instead any post-learning similarity rating bias would only reflect strategic judgment bias to rate faces as more similar due to the same category label (total bias = strategic judgment bias).

### Procedure

Experimental procedures were identical for participants in both conditions and consisted of four phases: passive viewing, pre-learning similarity ratings, feedback-based category learning, and post-learning similarity ratings. Each phase is detailed below.

#### Passive viewing

Prior to any learning, participants were presented with each of the nine face-blends to familiarize them with the stimuli and the degree of similarity between all faces before collecting perceived similarity ratings. Each of the nine training blends were viewed once (3s), in random order, without any labels and without making any responses. Trials were separated by a fixation inter-stimulusinterval (ISI; 1s).

#### Pre-learning similarity ratings

Prior to learning, all possible 36 pairwise comparisons of the nine training blends were presented and participants rated the similarity of the two faces on a scale from one to six (1 = two faces appear very dissimilar, 6 = two faces appear very similar). Trials were presented for 6s with a fixation ISI (1s) after each response.

#### Feedback-based category learning of families

On each trial, a single face was presented on the screen for 4s along with three family surnames (Miller, Wilson, Davis) as response options. Participants were instructed to indicate family membership via button press. After each response, corrective feedback was presented (2s), followed by a fixation ISI (1s). Participants completed five blocks of category learning separated by a self-paced break. Each block consisted of three repetitions of each stimulus, for a total of 15 repetitions per stimulus across the entire category learning phase.

#### Post-learning similarity ratings

Perceived similarity ratings were again collected immediately after the learning phase with the same procedure as the pre-learning ratings. Pairwise comparisons of the faces were presented in a different random order than the pre-learning ratings.

### Statistical analyses

#### Category learning

To determine how well participants learned the category labels in both conditions, we examined learning changes across time using a 2 [category structure condition (similarity-consistent, similarity-inconsistent)] x 5 [learning phase (block 1, block 2, block 3, block 4, block 5)] repeated measures ANOVA with learning phase as a within-subjects factor. Greenhouse-Geisser corrections for any violations of sphericity as detected via Mauchly’s test are noted as GG in results.

#### Category bias

To establish that the blending procedure successfully manipulated the physical similarity and perceived similarity of the faces, we first compared the pre-learning similarity ratings for faces that shared a blending parent and faces that did not share a blending parent, collapsed across conditions, using a paired t-test. We predicted that faces that shared a parent would be rated as more similar than faces that did not share a parent (Figure 2A) indicating that participants are sensitive to the similarity structure among the blended stimuli.

Of primary interest was the measure of category bias in perceived similarity ratings *after* learning (Ashby et al., 2020; Ashby and Zeithamova, 2022; Bowman et al., 2022). We sought to compare the degree of category bias when there are category-relevant features that can be extracted with the degree of category bias when there are no category-relevant features that can be extracted. Specifically, one of the pre-learning trial types (shared parent or no shared parent) can be further split after learning based on category membership, with the resulting trial types unique to each experimental condition (Figure 2B). In the similarity-consistent condition, the shared-parent trial type can be further split into two: faces that share a parent and have the same family name and faces that share a parent but have a different family name (Figure 2B, left panel, blue bars). Category bias in this condition was calculated from these shared-parent trial types by taking the difference of the similarity ratings for pairs of faces that were from the same family and similarity ratings for pairs of faces from different families (Figure 2B, left panel, difference between blue bars). Note that in the similarity-consistent condition, the no-shared-parent trials cannot be split into same and different category pairs because in this category structure only faces that shared a blended parent belonged to the same category. Thus, all faces that do not share a parent belong to different categories in this condition (see colored boxes illustrating categories determined across rows in Figure 1A).

In the similarity-inconsistent condition, the no-shared-parent trial type can be further split into two: faces that do not share a parent but belong to the same family and faces that do not share a parent and belong to different families (Figure 2B, right panel, red bars). Category bias in this condition was calculated from these no shared parent trial types by taking the difference of the similarity ratings for pairs of faces that were from the same family and similarity ratings for pairs of faces from different families (Figure 2B, right panel, difference between red bars). Critically, in the similarity-inconsistent condition the shared-parent trials cannot be split into same and different category pairs because in this category structure only faces that **did not share** a blended parent belong to the same category. Thus, all faces that share a parent belong to different categories in this condition (Figure 1B). Importantly, the category bias in both conditions compares similarity ratings for faces that belong to the same category with ratings for faces that are equated for physical similarity but belong to different categories.

We first examined category bias (same category minus difference category) in each condition separately using a one-sample *t*-test to determine whether category bias is significantly greater than zero. To examine the relative contribution of perceptual change and strategic processes on similarity ratings, we then compared post-learning category bias between conditions using an independent samples *t*-test. The same measures of category bias were also extracted for the same pairs of faces from pre-learning similarity ratings and compared to post-learning category biases using a paired *t*-test. This allowed us to confirm that category biases measured after learning were driven by learning and not capturing potential random pre-existing biases. In addition to test statistics and *p*-values, we include the effect sizes and their confidence intervals throughout the results.

## Results

### Category learning performance

To examine changes in category learning performance across time, mean categorization accuracy was calculated for each of the five blocks of the feedback-based category learning task (Figure 3). Categorization accuracy scores were submitted to a 2 x 5 repeated measures ANOVA, with experimental condition (similarity-consistent, similarity-inconsistent) as a between-subject factor and learning phase (block 1, block 2, block 3, block 4, block 5) as a within-subject factor. A significant main effect of condition [F(1, 207) = 113.48, *p* < 0.001, *η_p_*^2^ = 0.354] indicated participants had higher category-learning performance when faces shared physical features [M_accuracy_ = 67%, 95% CI (64%, 70%), chance = 33%] compared to when faces did not share any physical features [M_accuracy_ = 44%, 95% CI (41%, 47%), chance = 33%]. A significant main effect of learning phase [F(2.85, 590.33) = 151.67, *p* < 0.001, *η*^2^*_p_* = 0.42, *GG*] with a significant linear trend [F(1, 207) = 288.70, *p* < 0.001, *η_p_*^2^ = 0.582] indicated increased accuracy as learning progressed. Lastly, a significant interaction between experimental condition and learning phase [F(2.85, 590.33) = 3.92, *p* = 0.010, *η_p_*^2^ = 0.019, *GG*] indicated differences in the learning rate between conditions. Follow-up comparisons of the two conditions on each block showed that while the differences in performance were large and significant throughout (all t > 7.0, *p* < 0.001, *d* > 0.97 in each block), the effect size fluctuated among blocks. The largest difference in performance between conditions was observed in Block 1 of training (*d* = 1.39) and the smallest difference in Block 5 (*d* = 0.97). As suggested by Figure 3, performance in the similarity-inconsistent condition continued to improve through the fifth block of training while improvements slowed down earlier in the similarity-consistent condition.

Overall, results from the training phase indicated that participants learned similarity-consistent categories to a higher degree than similarity-inconsistent categories. Namely, a lack of shared physical features between same-family faces made the learning task more difficult for participants in the similarity-inconsistent condition. Due to the low learning performance, measuring and interpreting category bias is a challenge in this condition. For example, if a category bias is not found in the similarity-inconsistent condition, it may argue against a contribution of strategic judgment bias or instead reflect a lack of opportunity to show a strategic bias because participants were not able to adequately learn the family labels. Thus, we conducted all the following analyses twice. In addition to analyses with the full category-inconsistent sample, we also focused on a subset of participants from the similarity-inconsistent condition whose final training block performance was above 60%. This resulted in a subset (*n* = 35) whose average final training performance matched that from the similarity-consistent condition (see the dashed line in Figure 3). We refer to this subset as “similarity-inconsistent (memory-matched)”. Notably, using the memory-matched subset cannot eliminate the inherent difficulty differences across conditions, as evidenced by longer reaction times during the last block of training in the similarity-inconsistent (memory-matched) group (M_correctRT_ = 1.67) compared to the similarity-consistent group [M_correctRT_ = 1.41; t(137) = 4.29, *p* < 0.001, *d* = 0.84]. However, analyzing the subset of good learners in the similarity-inconsistent condition allows us to make sure that any potential differences in category bias are not driven simply by a lack of memory for the category labels in that condition.

### Category bias in similarity ratings

First, we examined similarity ratings prior to learning category information collapsed across conditions, to verify that participants were sensitive to the similarity manipulation. Results presented in Figure 4A indicated that faces which shared a parent were rated as more similar than faces that did not share a parent [M_diff_ = 1.39; t(208) = 31.56, *p* < 0.001, *d* = 2.18, 95% CI (1.93, 2.43)], confirming that our blending procedure worked as intended. As a secondary check, we examined whether there was evidence of a pre-existing bias toward category-membership prior to learning. As anticipated, there was no evidence for a category bias in perceptual similarity ratings prior to category learning in either condition [similarity-consistent: t(103) = 0.07, *p* = 0.47, *d* = 0.007, 95% CI (−0.19, 0.20); similarity-inconsistent: t(104) = −0.13, *p* = 0.45, *d* = −0.013, 95% CI (−0.20, 0.18)].

To measure the emergence of a category bias after learning, we first examined category bias in post-learning similarity ratings in each condition. In line with prior work, we found evidence for a post-learning category bias in the similarity-consistent condition (Figure 4B, left panel, significant difference between blue bars; t(103) = 4.47, *p* < 0.001, *d* = 0.44, 95% CI (0.24, 0.64)]. A paired-samples t-test confirmed that the category-bias observed post-learning was not a result of pre-existing differences in perceived similarity ratings prior to learning [post-bias vs. pre-bias M_diff_ = 0.54; t(103) = 4.72, *p* < 0.001, *d* = 0.46, 95% CI (0.26, 0.66)]. In comparison to pre-learning, participants increased their similarity rating for faces that shared a blended parent but belonged to the same family category [t(105) = 1.86, *p* = 0.066, *d* = 0.18] and decreased their similarity rating for faces that shared a blended parent but belonged to different family categories [t(105) = −4.30, *p* < 0.001, *d* = −0.42]. Participants who showed better category learning during training showed stronger post-learning category bias [R^2^ = 0.27, F(1, 102) = 37.71, *p* < 0.001], consistent with the notion that the bias is driven by category learning.

No evidence for a category bias was found in the similarity-inconsistent condition, neither in the full sample [Figure 4B, middle panel, no difference between red bars; t(104) = −0.82, *p* = 0.207, *d* = −0.08, 95% CI (−0.27, 0.11)] nor in the memory-matched sub-sample [Figure 4B, right panel, no difference between red bars; t(34) = 0.16, *p* = 0.44, *d* = 0.027, 95% CI (−0.30, 0.36)]. Consistent with the lack of bias among the top learners, there was also no relationship between how well participants learned the category labels during training and their subsequent category bias [R^2^ = 0.01, F(1, 103) = 1.13, *p* = 0.29], as there was little bias among both good and poor learners. Thus, even when controlling for memory for category labels, a category-bias in post-learning similarity ratings was only evident for categories where members shared physical features. No evidence for category bias was observed when stimuli within a category did not share features, indicating that without a physical similarity structure, a shared category label is not sufficient to induce a bias in perceived similarity ratings.

## Discussion

Categorization tasks utilizing artificial or photography-based faces have decades of tradition (Etcovv and Magee, 1992; Calder et al., 1996; Young et al., 1997; Stevenage, 1998; Goldstone and Steyvers, 2001; Goldstone et al., 2001; Roberson et al., 2007; Ashby et al., 2020; Ashby and Zeithamova, 2022; Bowman et al., 2022). Here, we utilized blended faces because it allowed us to control the similarity structure of the categories while offering stimuli that are realistic looking and holistically perceived. We sought to determine the degree to which perceived similarity ratings in the face-blend categorization task used in our prior work (Ashby et al., 2020; Ashby and Zeithamova, 2022; Bowman et al., 2022) are driven by perceptual and strategic judgment biases. We tracked post-learning category bias after a traditional feedback-based category learning task under two learning conditions. In the similarity-consistent condition, category membership was consistent with the similarity structure among stimuli where category bias can reflect a combination of a perceptual bias and a strategic judgment bias. In the similarity-inconsistent condition, category membership was orthogonal from physical features and any similarity rating bias could be attributed to a strategic judgment bias alone. Results showed evidence for a category bias in post-learning similarity ratings, but only when items shared physical features within categories. Thus, we replicated the findings of categorization-driven biases, but also identified a boundary condition for this effect, indicating that its emergence is conditioned on the existence of a similarity structure within categories. Because no category bias was observed in the condition where items shared labels but did not share physical characteristics, the emergence of a category bias in similarity ratings appears to largely reflect a change in perceived similarity and not merely a strategic decision to rate items with shared labels more similarly.

Our new approach demonstrating a role of perceptual changes in category-biased similarity ratings provides further support for an attentional theory of category learning and category-induced biases. Past work has postulated that learning category information prompts individuals to shift their attention toward category-relevant features and away from category-irrelevant features (Nosofsky, 1986, 1987), which leads to changes in perception to highlight within-category similarity and between category differences (Goldstone, 1994a; Beale and Keil, 1995; Kurtz, 1996; Livingston et al., 1998; Goldstone and Steyvers, 2001; Gureckis and Goldstone, 2008; Folstein et al., 2013a,b; Soto, 2019). For example, when one acquires a new category where category membership depends on brightness but not size, increased attention to brightness “stretches” that dimension, enhancing perception of differences in brightness. In contrast, because size is irrelevant for category membership, decreased attention to the size dimension “shrinks” the perceptual space along the size dimension, decreasing perception of size differences (Goldstone, 1994a). Within the context of the current task, imagine that one of the category-relevant parents might have prominent eyebrows but the three relevant parents do not differ much in their mouth shape. The increased attention to eyebrows and decreased attention to mouth shape after category learning would lead participants to be more likely to perceive eyebrow differences (highlighting between-category differences and within-category similarity of eyebrows) and less strongly perceive mouth differences (making any within-category differences of mouth shape driven by irrelevant parent features less salient). Because faces are processed differently by the brain compared to other types of objects (for reviews see Ruiz-Soler and Beltran, 2006; Tsao and Livingstone, 2008; Behrmann et al., 2016) and face perception may be “special” in that faces are processed on a more holistic level than other types of stimuli (Farah et al., 1998; *for review see* Piepers and Robbins, 2012), it will be important to test generalization of current findings to other types of stimuli. Nevertheless, as category-driven attentional biases have been previously shown for many types of stimuli (Bornstein et al., 1976; Bornstein and Korda, 1984; Livingston et al., 1998; Goldstone and Steyvers, 2001; Franklin and Davies, 2004), the principles of how category learning biases perception through stretching and shrinking perceptual space along different dimensions may be relatively universal.

Our behavioral data suggesting a bias in perception, consistent with shifts of attention, further align with evidence for an attentional shift as well as reports of category bias reflected in the brain. For example, Mack et al. (2013) found neural representations for category features distributed widespread across the cortex but only when attention weights to individual stimulus features were considered. Ashby and colleagues (2020) found neural biases toward category-relevant information widespread throughout the cortex when items shared physical features. The current study contributes further support for the attentional theory of category bias by demonstrating that category bias only emerges under conditions where items within categories share physical features, giving individuals category-relevant features to which they may attend.

Although our findings are consistent with the proposals that category learning results in attentional shifts from category irrelevant to relevant features, creating perceptual biases, we cannot completely rule out the possibility that strategic bias may still contribute to the total bias observed for similarity-based categories. For example, fast category learning in the similarity-consistent condition may have allowed participants to engage in attentional tuning to category-diagnostic features more extensively (Blair et al., 2009; Chen et al., 2013; McColeman et al., 2014), which in turn may have facilitated faster access to the associated category labels during the rating task and greater impact of labels in ratings. The differences in reaction times between conditions, even when limited to the top learners in the similarity-inconsistent condition, indeed indicate that the label became available to participants much faster in the similarity-consistent than the similarity inconsistent condition. Furthermore, the similarity-inconsistent condition required rote memorization of face-label pairs, which may not be sufficient to evoke a strategic bias. For example, Fotiadis and Protopapas (2022) found facilitatory effects of verbal labels on category learning but **not** for a paired-associate task that did not involve similarity-based categories which may more closely resemble the similarity-inconsistent condition in the current study. As category labels may not have appeared as meaningful information in the similarity-inconsistent condition, the effect of the shared label may have been minimal on both perceptual and strategic judgments when it was not strongly activated by the stimulus (Lupyan, 2012). Conceptually consistent with the Fotiadis and Protopapas (2022) study, our findings demonstrate that shared category labels, *per se*, are not sufficient to create category biases in similarity ratings when categories do not align with similarity structure, providing a new boundary condition on the effect of language on perception.

Our findings indicated that the role of strategic judgment in perceived similarity ratings in the current task is likely limited. In contrast, Goldstone and colleagues (2001) concluded that category bias in similarity ratings measures not only the perceptual similarity of the items themselves but also the similarity of their associated labels and categories. One reason for the differences in findings between Goldstone and colleagues (2001) and the current study may be differences in experimental design. First, a category bias (greater within- vs. between-category similarity) was already present prior to learning in the Goldstone et al. (2001) study because items within a category tended to be more similar than items from different categories. Here, we only compared items equated for physical similarity within and between categories to ensure that the category bias, and subsequent changes in perceived similarity, are due to category learning alone and cannot be explained by pre-existing similarity differences. In fact, participants in our similarity-inconsistent condition had to work *against* the similarity structure among the stimuli to learn the category assignments. Second, category bias that reflected a strategic judgment bias in the previous study was measured indirectly through similarity ratings to a separate, non-categorizable, neutral face. Furthermore, participants were trained to learn category information under conditions where shared similarity within and between categories was present in varying degrees. In contrast, we trained participants to learn category information using a category similarity structure that held similarity constant within and between categories (i.e., similarity-consistent group) or held similarity orthogonal to within and between category designation (i.e., similarity-inconsistent group), allowing for a direct and clear measurement of category bias. Finally, the current study is the first to our knowledge that measures the degree of strategic judgment bias that may be inherent to similarity ratings by including a comparison learning condition where physical similarity is orthogonal to category structure and associated category labels. Nevertheless, it will be important to explore the strategic bias question in the future with other paradigms, including arbitrary or “functional” categories where stimuli with no inherent similarity structure are arbitrarily assigned to categories (Pan and Sakagami, 2012; Taylor et al., 2021), to fully determine the extent to which verbal labels influence similarity judgments across a broader range of category structures.

In our prior behavioral and neuroimaging work, we found evidence for category biases in face similarity ratings even when controlling for physical similarity (Ashby et al., 2020; Ashby and Zeithamova, 2022; Bowman et al., 2022). These findings suggest that when a degree of shared similarity exists and a verbal label aligning with some of those shared features exists, the brain may be hardwired to allocate a disproportionate amount of attention to shared features. This effect may be one of the mechanisms behind perpetuating social categories and stereotypes (Amodio, 2014). For example, racial biases may be driven by some physical similarities within groups and differences between groups that are then overexaggerated in people’s perceptions (Byatt and Rhodes, 2004). Objectively, the degree of variability across facial features within various races is small (Goldstein, 1979a,b). However, when individuals differ along some physical feature (such as skin color) and a category label (such as race) becomes associated with that feature, the feature can become emphasized in people’s perceptions. In contrast, any features not aligned with category membership become less perceptually salient or ignored. This would alter our perceptions, highlighting within-category similarity and between-category differences, and biasing us to perceive the categories as more differentiated than they really are.

Interestingly, we also found that shared features were necessary to induce a category bias in perception in the current study. Yet, not all biases in the real world, such as religious bias or political affiliation bias, can be tied to salient physical features. Therefore, these results may indicate that different mechanisms could be responsible for types of in-group/out-group biases that are not grounded in physical similarity. Rather than relying on attentional biases to category relevant features, biases that are not similarity-based may work through associative inference, where value or affect associated with one member of a category spreads to other members of the category through associations (Pan and Sakagami, 2012; Wimmer and Shohamy, 2012; Martinez et al., 2016). More broadly, our results indicate that category learning involving similarity-based structure and learning involving arbitrary category labels likely involve distinct mechanisms.

## Figures and Tables

**FIGURE 1:**
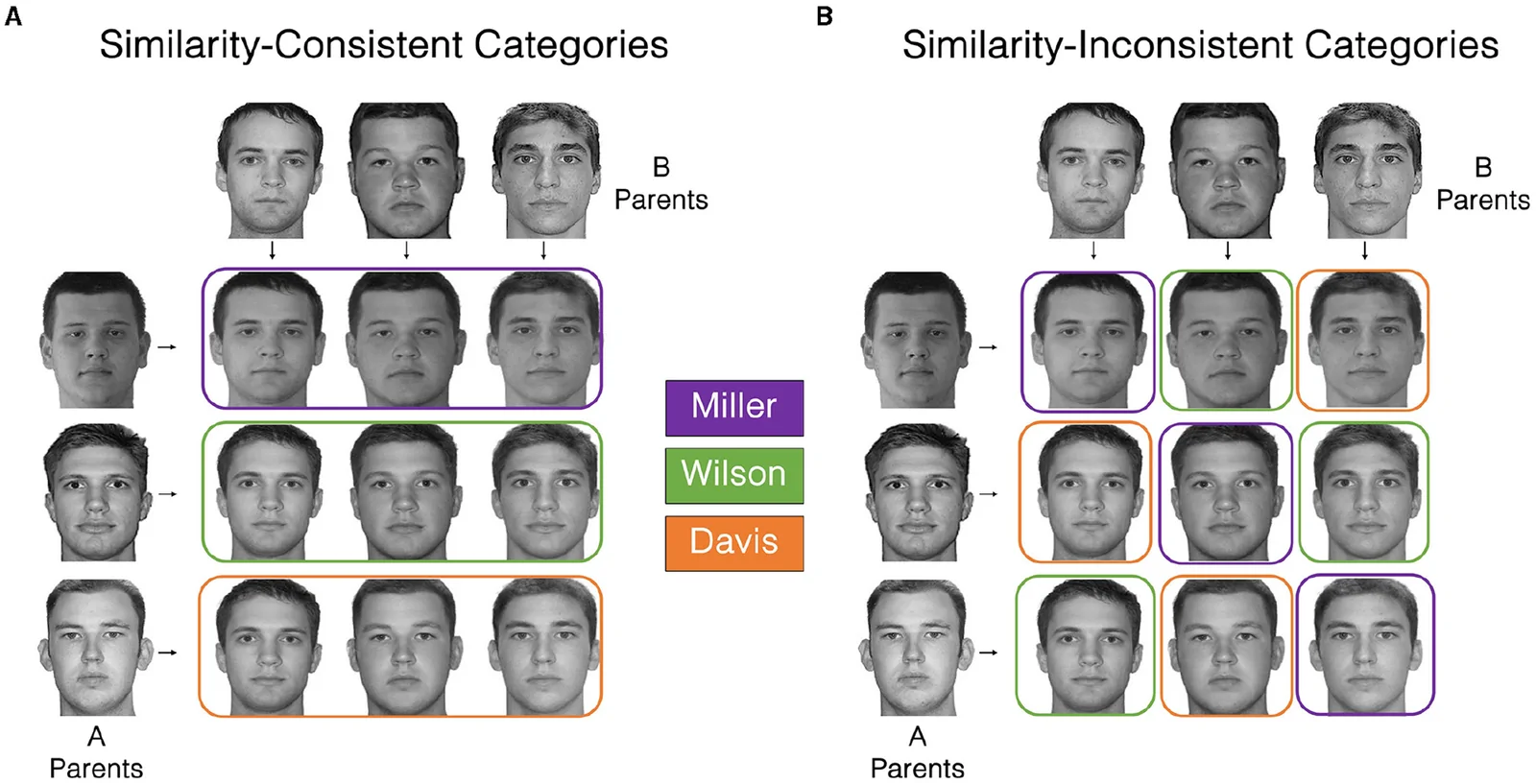
Face-blend stimulus structure by experimental condition. **(A)** Similarity-consistent categories. Blends of each “A” parent face share the same category label (family surname) and share physical features of the parent face. Blends of each “B” parent face (faces in each column) also share physical features but not category label. **(B)** Similarity-inconsistent categories where category membership is orthogonal to physical similarity. Blends of the same parent face never belong to the same category. Reprinted and adapted (with permission) from the Dallas Face Database (Minear and Park, 2004) and the CVL Face Database (Peer, 1999).

**FIGURE 2:**
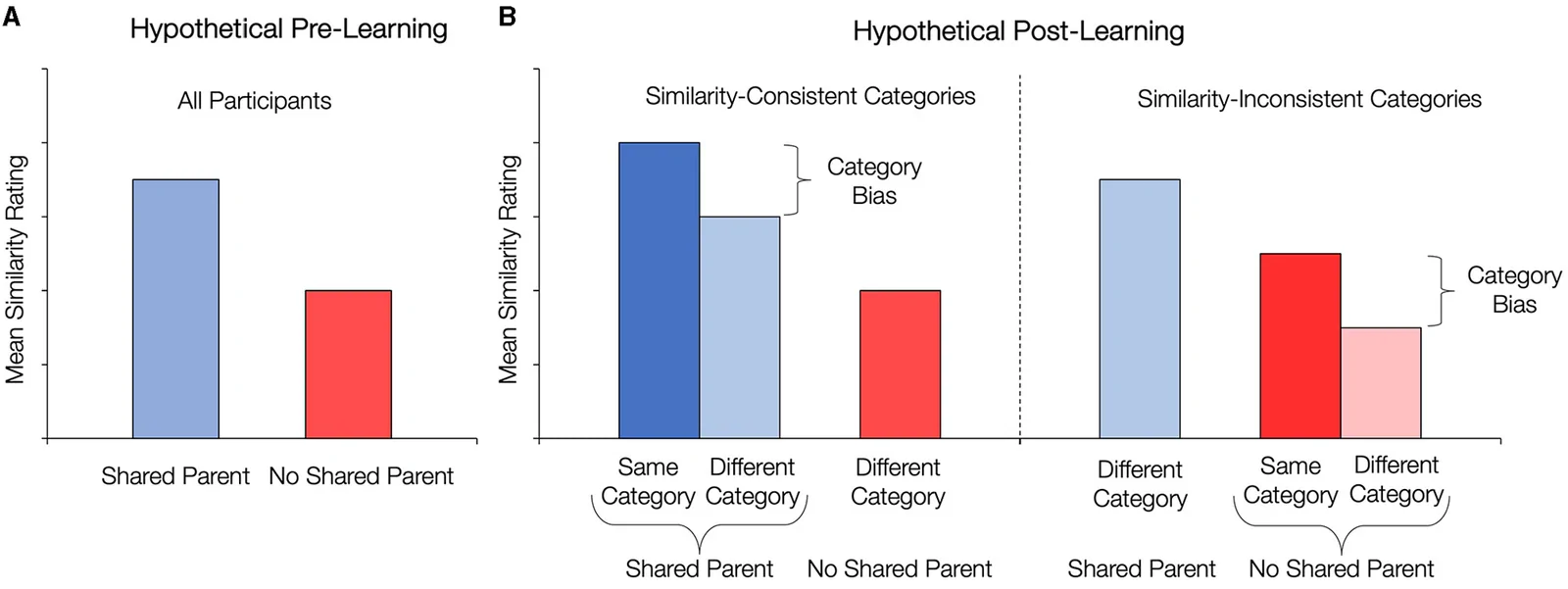
Hypothetical outcomes illustrating the manipulation check pre-learning and the calculation of category bias in each condition post-learning. **(A)** Prior to learning category information, and regardless of condition, faces that share a parent are predicted to be rated as more similar than faces that do not share a blended parent. **(B)** Hypothetical results showing category bias in each condition. For similarity-consistent categories, some pairs of faces that share a parent belong to the same category while other pairs of faces that share a parent belong to different categories. Category bias is the difference in post-learning similarity ratings for those two types of similarity rating trials. For similarity-inconsistent families, some pairs of faces that do *not* share a parent belong to the same category while other pairs of faces that do *not* share a parent belong to different categories. Category bias is the difference in post-learning similarity ratings for those two types of similarity rating trials.

**FIGURE 3:**
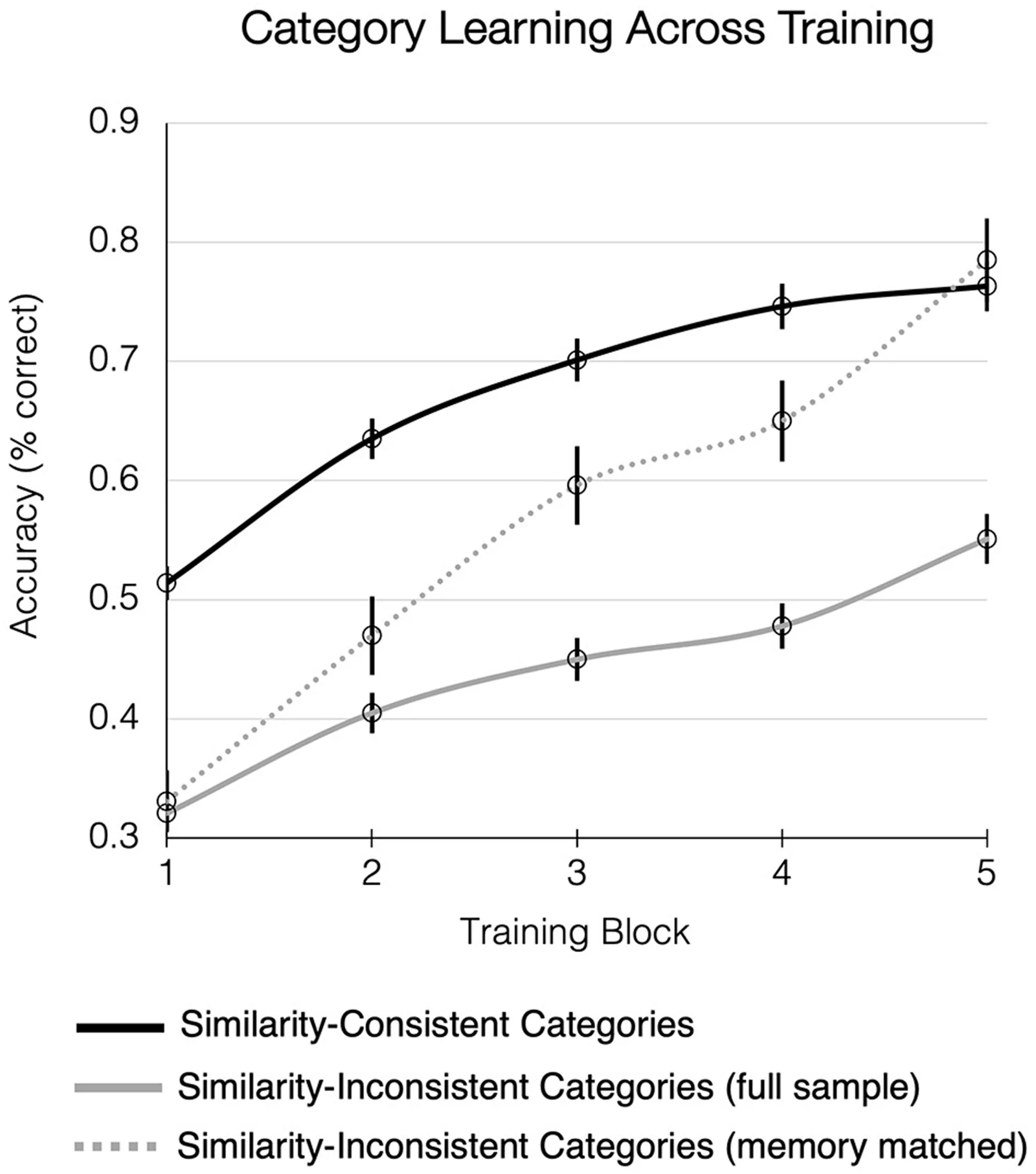
Category learning performance across training for the similarity-consistent condition (dark line) and similarity-inconsistent condition (solid gray line). Category learning was more difficult when category members did not share physical features as indicated by lower performance in the similarity-inconsistent condition. Also included is performance from a subset of the similarity-inconsistent group (dashed gray line) whose categorization performance matched the category-consistent group by the end of training. Chance = 33% for three categories.

**FIGURE 4:**
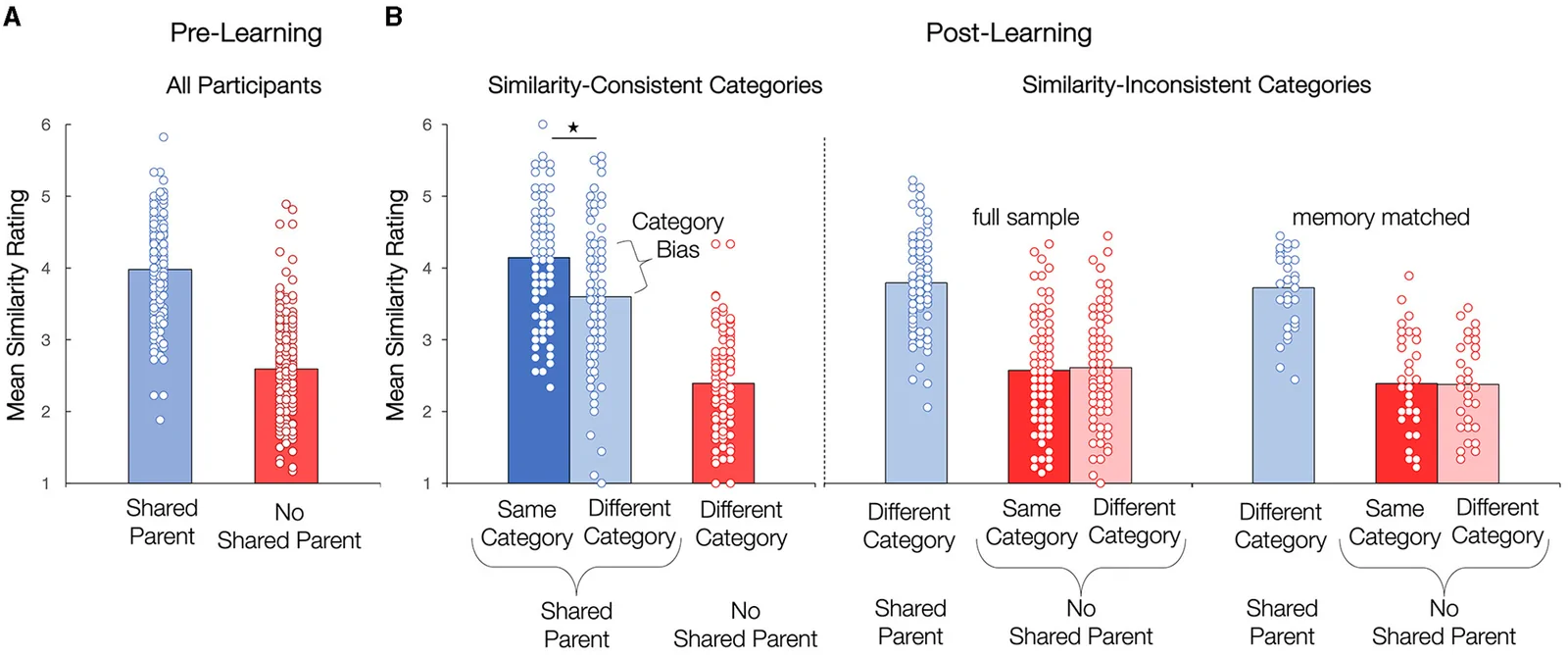
Observed perceived similarity ratings before and after learning. **(A)** Pre-learning similarity ratings collapsed across conditions. Faces that share a parent are rated as more similar than faces that do not share a parent. **(B)** Post-learning similarity ratings for the similarity-consistent and similarity-inconsistent conditions. Ratings for the similarity-inconsistent condition are presented for the full sample as well as a memory-matched sub-sample. There is evidence for a category bias in post-learning ratings in the similarity-consistent category condition (significant difference in blue bars) but not the similarity-inconsistent categories condition (no difference in red bars in either sample). *p < 0.05.

## Data Availability

The datasets presented in this study can be found in online repositories. The names of the repository/repositories and accession number(s) can be found in the article/supplementary material.
